# Self-Collected Mid-Turbinate Swabs for the Detection of Respiratory Viruses in Adults with Acute Respiratory Illnesses

**DOI:** 10.1371/journal.pone.0021335

**Published:** 2011-06-23

**Authors:** Oscar E. Larios, Brenda L. Coleman, Steven J. Drews, Tony Mazzulli, Bjug Borgundvaag, Karen Green, STOP-Flu Study, Allison J. McGeer

**Affiliations:** 1 Departments of Medicine and Laboratory Medicine, Royal University Hospital, Saskatoon, Saskatchewan, Canada; 2 Faculty of Medicine, University of Saskatchewan, Saskatoon, Saskatchewan, Canada; 3 Department of Microbiology, Mount Sinai Hospital, Toronto, Ontario, Canada; 4 Faculty of Medicine, University of Toronto, Toronto, Ontario, Canada; 5 Department of Microbiology, Immunology & Infectious Diseases, University of Calgary, Calgary, Alberta, Canada; 6 Department of Microbiology, Provincial Laboratory for Public Health, Calgary, Alberta, Canada; 7 Department of Emergency Medicine, Mount Sinai Hospital, Toronto, Ontario, Canada; University of Georgia, United States of America

## Abstract

**Background:**

The gold standard for respiratory virus testing is a nasopharyngeal (NP) swab, which is collected by a healthcare worker. Midturbinate (MT) swabs are an alternative due to their ease of collection and possible self-collection by patients. The objective of this study was to compare the respiratory virus isolation of flocked MT swabs compared to flocked NP swabs.

**Methods:**

Beginning in October 2008, healthy adults aged 18 to 69 years were recruited into a cohort and followed up for symptoms of influenza. They were asked to have NP and MT swabs taken as soon as possible after the onset of a fever or two or more respiratory symptoms with an acute onset. The swabs were tested for viral respiratory infections using Seeplex® RV12 multiplex PCR detection kit. Seventy six pairs of simultaneous NP and MT swabs were collected from 38 symptomatic subjects. Twenty nine (38%) of these pairs were positive by either NP or MT swabs or both. Sixty nine (91%) of the pair results were concordant. Two samples (3%) for hCV OC43/HKU1 and 1 sample (1%) for rhinovirus A/B were positive by NP but negative by MT. One sample each for hCV 229E/NL63, hCV OC43/HKU1, respiratory syncytial virus A, and influenza B were positive by MT but negative by NP.

**Conclusions:**

Flocked MT swabs are sensitive for the diagnosis of multiple respiratory viruses. Given the ease of MT collection and similar results between the two swabs, it is likely that MT swabs should be the preferred method of respiratory cell collection for outpatient studies. In light of this data, larger studies should be performed to ensure that this still holds true and data should also be collected on the patient preference of collection methods.

## Introduction

Viruses account for approximately 80% of acute respiratory diseases in developed countries [1]. Ciliated epithelium in the posterior nasopharynx is the site of infection for most of these viruses and the accepted standard diagnostic specimen for viral detection in adults has become the NP swab [2]. Healthcare providers must be trained in the collection of NP swabs and their collection is uncomfortable for the patient.

Virus detection from NP swabs has traditionally been performed using a combination of rapid antigen tests, and conventional and rapid cell culture techniques [3], [4]. The sensitivity of these tests is suboptimal and newly discovered viruses cannot be detected by rapid antigen tests. Nucleic acid amplification tests have now been developed for respiratory pathogens and have the ability to simplify testing into a single multiplex polymerase chain reaction (PCR) test [1].

Copan Diagnostics Inc. recently introduced flocked swabs containing nylon fiber strands designed to more rapidly absorb respiratory epithelial cells and more efficiently release them into transport media [5], [6]. Flocked mid-turbinate swabs are an alternative to NP swabs that are more comfortable for patients and may permit self-collection by adult patients. Recent literature has shown that the sensitivity and specificity of pernasal flocked swabs were 98.5% and 100%, respectively when compared to NP aspirates for respiratory virus detection in children [7].

We hypothesized that increased sensitivity and specificity of PCR for respiratory virus detection combined with the increased sensitivity associated with flocked swabs would make self-collected mid-turbinate swabs an acceptable alternative to nasopharyngeal swabs for the diagnosis of respiratory viral infection in adults. The objective of this prospective study was to compare respiratory virus identification in self-collected flocked mid-turbinate swabs to nurse-obtained flocked NP swabs in adults.

## Methods

The study was approved by Health Canada and the Research Ethics Board of Mount Sinai Hospital with written informed consent obtained from all participants. In October 2008, 60 working adults from Toronto, Ontario were recruited into a pilot study of influenza infection and followed for symptoms of acute respiratory illness until May 2009. Subjects were asked to report all episodes with either fever or two or more respiratory symptoms (stuffy/runny nose, cough, scratchy/sore throat, sneezing, hoarseness) with acute onset (i.e. not associated with allergies). They were then to submit a self-collected flocked mid-turbinate swab (Copan FLOQSwabs™; Copan, Italy) and have a nurse-performed flocked NP swab obtained as soon as possible, preferably within 24–48 hours, after onset of symptoms.

Subjects were provided with a mid-turbinate swab kit (including written and pictorial instructions), they were also verbally instructed to insert the mid-turbinate swab into one outer nare up to the swab's collar and rotate the swab three times, then place it in universal transport media. Study nurses were available to answer questions. Mid-turbinate swabs were collected immediately before NP swabs, which were obtained from the opposite nare by trained study nurses. Swabs were transported to the Ontario Central Public Health Laboratory in universal transport medium (UTM-RT; Copan, Italy).

Total nucleic acid was extracted from each specimen using the easyMAG® (bioMerieux, Quebec, Canada) automated extraction system. To control for extraction, all specimens were tested for human *gapdh* using the TaqMan® GAPDH Control RT-PCR Kit (Applied Biosystems, Foster City, USA) according to manufacturer's instructions. Total nucleic acid was tested using the Seeplex® RV12 multiplex PCR detection kit (Seegene, Seoul Korea) for influenza A and B, respiratory syncytial virus (RSV) A and B, parainfluenza virus (PIV)1, PIV2, PIV3, human coronavirus (hCoV) 229E/NL63, hCoV OC43/HKU1, rhinovirus A/B, adenovirus and human metapneumovirus. PCR products were visualized using ethidium bromide on 2% agarose electrophoresis gels as previously described [8]. Although specimens were identified by code only, it was not possible to blind laboratory personnel to the swab type during processing.

Statistical analysis was completed using Prism Windows 5.03 (GraphPad Software Inc., La Jolla, USA).

## Results

**Figure 1:**
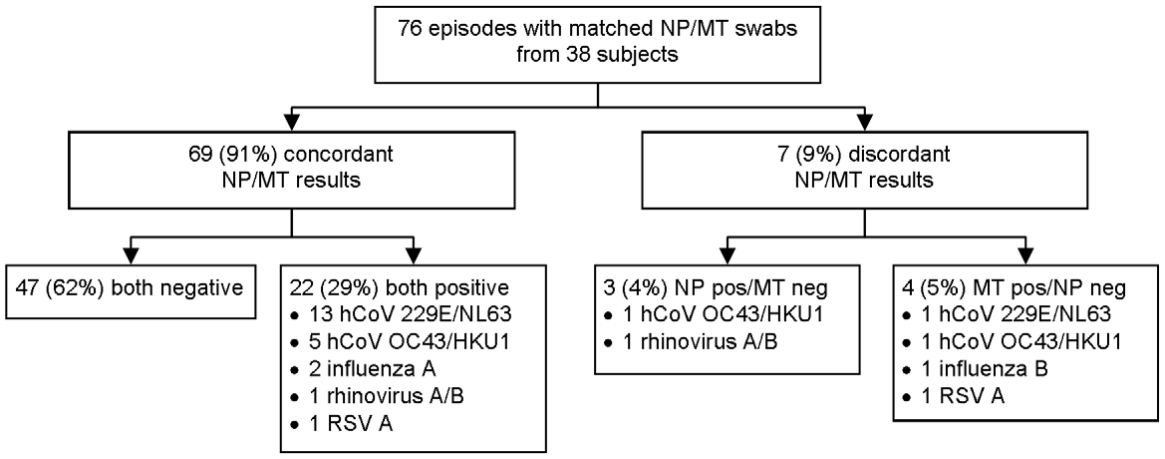
Results from matched nurse-collected nasopharyngeal and self-collected mid-turbinate swabs from adults with acute respiratory illness. Foot note: hCoV: human coronavirus, MT: mid-turbinate, neg: negative, NP: nasopharyngeal, pos: positive, RSV: respiratory syncytial virus.

Thirty eight subjects reported 76 episodes with acute respiratory symptoms for which matched nurse-collected NP and self-collected mid-turbinate (MT) swabs were collected on the same day (Figure 1). The median age of the 60 participants was 41.1 years (range 23–59 years), 43 (72%) were female, 9 (15%) were smokers, and 12 (20%) had an underlying medical conditions (most commonly diabetes mellitus (3) and hypothyroidism (3)). Fifty four participants worked in a healthcare facility with 25 (42%) providing direct patient care. There were no differences in demographic or medical characteristics between subjects who submitted matched sets of swabs and those who did not or between subjects who had a specimen yielding a respiratory virus and those who did not (data not shown). Subjects submitted from 1 to 7 matched sets of swabs for testing over the study period (19 submitted 1, 10 submitted 2, 4 submitted 3, 2 submitted 4, 2 submitted 5, and 1 submitted 7).

Of the 76 matched sets of swabs submitted, 33 (43%) were submitted on the first day of illness, 28 (37%) on the second, 9 (12%) on the third day, and the remaining 6 (8%) on days 4–9. Eight of 33 (24%) swabs obtained on the first day of illness yielded a virus, compared to 20 of 37 (54%) obtained on days 2 or 3, and 1 of 6 (17%) obtained subsequently (P = 0.02). Viruses were somewhat more likely to be detected from persons reporting febrile illness (7/14 (50%) episodes with fever yielded viruses versus 22/62 (35%) afebrile episodes (P = 0.48). Of episodes with credible duration information, illnesses of longer duration were somewhat more likely to have viruses detected with viruses detected in 11 of 39 (28%) episodes with a duration of symptoms of 3 or fewer days compared to 18 of 35 (51%) episodes with a duration of 4 days or longer (P = 0.057).

Overall, 29 of 76 (38%) if episodes had a virus identified by PCR: 22 (76%) identified by both NP and mid-turbinate swabs. The seven discrepant swabs included five different viruses (Figure 1). Considering a virus identified by PCR from either type of swab as valid, the sensitivity and negative predictive values of mid-turbinate and nasopharyngeal swabs are shown in Table 1.

**Table 1:** Sensitivity and negative predictive value of mid-turbinate swabs and nasopharyngeal swabs compared to combined results for the diagnosis of viral infection in adults with acute respiratory illness.

Respiratory virus	Total positive	Sensitivity(95% CI)^1^		Negative predictive value(95% CI)^1^	
		NP swab	MT swab	NP swab	MT swab
All	29	86% (73, 99)	90% (79, 100)	92% (82, 97)	94% (84, 98)
hCoV 229E/NL63	14	93% (68, 98)	100% (78, 100)	98% (92, 100)	100% (94,100)
hCoV OC43/HKU1	8	83% (44, 97)	71% (40, 93)	99% (92, 100)	97% (90, 99)
Influenza	3	67% (20, 93)	100% (40, 100)	99% (93, 100)	100% (95, 100)
Rhinovirus A/B	2	100% (29, 100)	50% (9, 91)	100% (95, 100)	100% (95, 100)
RSV A	2	50% (9, 91)	100% (29, 100)	99% (93, 100)	99% (93, 100)

Foot note: CI: confidence interval, hCoV: human coronavirus, MT: mid-turbinate, NP: nasopharyngeal, RSV: respiratory syncytial virus.

1 Not adjusted for dependence between observations (some swab pairings were from same subjects but collected at subsequent illness episodes).

## Discussion

These data suggest that self-collected, nylon, flocked mid-turbinate swabs may be an alternative to nurse-collected NP swabs for the detection of respiratory viruses by PCR in acute respiratory infections in adults. Our findings are similar to those reported by a study comparing parent and pediatrician collected mid-turbinate swabs in children (in which children much preferred the parent collected swab) [9] and to a number of other studies demonstrating only small differences between the yield of nasal swabs and NP aspirates in children when PCR was used for viral detection [7], [10]–[12]. However, NP aspirates have been shown, in other studies, to have a somewhat improved sensitivity compared to NP swabs in children, especially for RSV [13], and the power of our study is limited, particularly at the individual virus level. Our findings are somewhat limited in their generalizability in that our sample size was small and our participants were working-age adults, many of whom were healthcare providers. About 75% of the NP/MT swab pairs were collected from the same subjects but at subsequent illness episodes. If the subjects became more adept at self-collection of MT swabs, it may have affected the results. However, no pattern was noted for discrepant results for subjects with repeated collections. Given the results and limitations of this study, further research is needed to be confident that self-collected mid-turbinate swabs are adequate for all respiratory virus detection by PCR in adults.

Our findings are also compatible with previous data demonstrating peak viral shedding on the first or second day of acute respiratory illness [14]–[16] and studies that suggest that more severe illness is associated with higher concentrations of virus shed from the respiratory tract [14], [17].

The increased sensitivity of PCR for diagnosis may mean that mild acute viral respiratory illness can be accurately diagnosed in adults with self-collected mid-turbinate swabs. Our results, if confirmed, should facilitate both accurate diagnosis and studies of the epidemiology of viral respiratory illness in adults.
